# TAp63 contributes to sexual dimorphism in POMC neuron functions and energy homeostasis

**DOI:** 10.1038/s41467-018-03796-7

**Published:** 2018-04-18

**Authors:** Chunmei Wang, Yanlin He, Pingwen Xu, Yongjie Yang, Kenji Saito, Yan Xia, Xiaofeng Yan, Antentor Hinton Jr, Chunling Yan, Hongfang Ding, Likai Yu, Gang Shu, Rajat Gupta, Qi Wu, Qingchun Tong, William R. Lagor, Elsa R. Flores, Yong Xu

**Affiliations:** 1 Children’s Nutrition Research Center, Department of Pediatrics, Baylor College of Medicine, Houston, TX 77030 USA; 2 Molecular Physiology and Biophysics, Baylor College of Medicine, Houston, TX 77030 USA; 3 Brown Foundation Institute of Molecular Medicine, University of Texas Health Science Center at Houston, Houston, TX 77030 USA; 4 Department of Molecular Oncology, Cancer Biology and Evolution Program, Department of Cutaneous Oncology, H. Lee Moffitt Cancer Center, Tampa, FL 33612 USA; 5 Department of Molecular and Cellular Biology, Baylor College of Medicine, Houston, TX 77030 USA

## Abstract

Sexual dimorphism exists in energy balance, but the underlying mechanisms remain unclear. Here we show that the female mice have more pro-opiomelanocortin (POMC) neurons in the arcuate nucleus of hypothalamus than males, and female POMC neurons display higher neural activities, compared to male counterparts. Strikingly, deletion of the transcription factor, TAp63, in POMC neurons confers “male-like” diet-induced obesity (DIO) in female mice associated with decreased POMC neural activities; but the same deletion does not affect male mice. Our results indicate that TAp63 in female POMC neurons contributes to the enhanced POMC neuron functions and resistance to obesity in females. Thus, TAp63 in POMC neurons is one key molecular driver for the sexual dimorphism in energy homeostasis.

## Abstract (web summary)

Sexual dimorphism exists in a number of physiological processes, including energy homeostasis. Here, the authors show that pro-opiomelanocortin neurons in female mice fire more rapidly than males, and that deletion of the transcription TAp63 leads to a reduced neuronal firing rate and a male-like susceptibility to diet-induced obesity.

## Introduction

Sexual dimorphism exists in various physiological processes, including feeding behavior and energy homeostasis^1,2^. For example, total daily energy intake in male rats is higher than that in females, even when corrected by their larger lean body mass and metabolic rate^3^. In addition, high-fat diet (HFD) feeding leads to larger body weight gain in male rats/mice than in female counterparts^4–8^. While sex hormones (e.g., estrogens and testosterone) and the sex chromosome complement have been implicated as major contributors for these sex-related differences^9–11^, the cellular and molecular mechanisms underlying the sexual dimorphism in body weight balance remains to be fully revealed.

Pro-opiomelanocortin (POMC) neurons in the arcuate nucleus of hypothalamus (ARH) play essential roles in the regulation of feeding and body weight balance, as genetic ablation of POMC neurons in the ARH leads to hyperphagia and obesity in mice^12,13^. On the other hand, selective activation of ARH POMC neurons results in decreased food intake and body weight loss^13^, indicating that the neural activities of ARH POMC neurons are important for the regulation of energy homeostasis. In addition, transcription of the POMC gene itself is physiologically relevant for body weight control. One of the POMC gene products, α-melanocyte-stimulating hormone (α-MSH)^14^, acts upon brain melanocortin 3 and 4 receptors^15–18^ to suppress the food intake and body weight gain. Further, massive obesity is observed in mice with POMC gene deficiency^19^. Consistently, humans carrying loss-of-function mutations in the POMC gene develop obesity^20–22^. These findings indicate that normal POMC gene expression is fundamentally required to maintain normal body weight.

We observed that female mice have more POMC neurons in the ARH than males, and female POMC neurons display higher neural activities, compared to male counterparts. We generated mice with POMC-specific deletion or overexpression of TAp63, a transcriptionally active variant of p63 (a transcription factor). Systemic characterization of these mutant mouse models revealed that TAp63 contributes to sexual dimorphism in POMC neuron functions and regulates energy homeostasis in a sex-specific manner.

## Results

### Sexual dimorphism in POMC neurons

To explore the cellular basis for sexual dimorphism in energy balance, we first examined the neural activities of several neural populations that are known to play key roles in the regulation of energy homeostasis. These include ARH neurons co-expressing agouti-related peptide (AgRP) and neuropeptide Y (NPY)^23–25^, steroidogenic factor-1 (SF1) neurons in the ventromedial hypothalamic nucleus (VMH)^26^, single-minded 1 (SIM1) neurons in the medial amygdala (MeA)^27^, and SIM1 neurons in the paraventricular nucleus of the hypothalamus (PVH)^28^. We did not observe any sex-specific difference in resting membrane potential or firing rate of AgRP/NPY neurons, VMH SF1 neurons, or MeA SIM1 neurons (Supplementary Figure 1A-1C, 1E-1G). While male and female PVH SIM1 neurons showed similar resting membrane potential (Supplementary Figure 1D), the firing rate of female PVH SIM1 neurons was significantly lower than that of the male counterparts (Supplementary Figure 1H). However, since the majority of PVH SIM1 neurons are anorexigenic neurons that suppress food intake^29^, the decreased firing activity of female PVH SIM1 neurons should not account for the relative low body weight in female mice.

**Fig. 1:**
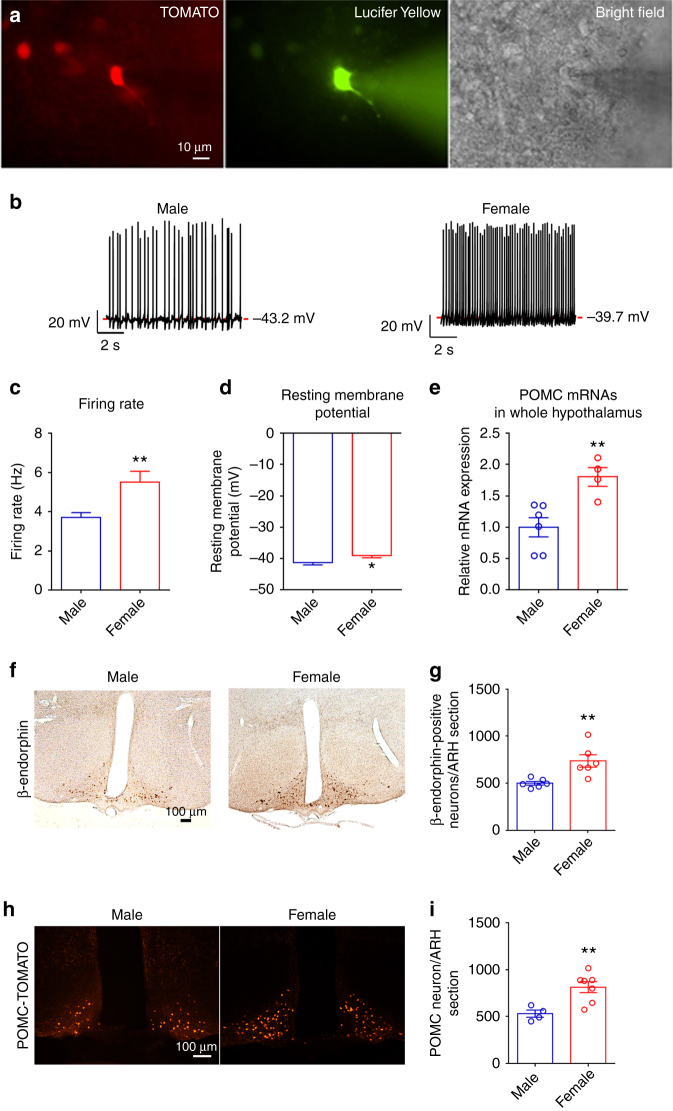
POMC neurons display sexual dimorphism in neural activities and gene transcription. **a** Fluorescence for TOMATO (left) and Lucifer Yellow (middle), and bright-field illumination (right) of the recorded POMC neuron in a brain slice prepared from *POMC-CreER*^*T2*^*/Rosa26-tdTOMATO* mouse with tamoxifen induction at 11 weeks of age. **b** Representative current clamp traces in POMC neurons from chow-fed male or female *POMC-CreER*^*T2*^*/Rosa26-tdTOMATO* mice at the age of 15–16 weeks. **c**, **d** Average firing rate (**c**) and resting membrane potential (**d**) in POMC neurons from male or female *POMC-CreER*^*T2*^*/Rosa26-tdTOMATO* mice. Data are presented as mean ± SEM. *N* = 37–78 per group. *P < 0.05 or **P < 0.01 in *t*-tests. **e** POMC mRNAs levels in the entire hypothalamus from male or female littermates (16 weeks of age), quantified by RT–qPCR. Data are presented as mean ± SEM with individual data points. *N* = 4 or 6 per group. **P < 0.01 in *t*-tests. **f** Representative immunostaining images for β-endorphin in the hypothalamus of male or female littermates (16 weeks of age). **g** Quantifications of β-endorphin-positive neurons in the hypothalamus from 1 out of five consecutive brain sections of male or female littermates. Data are presented as mean ± SEM with individual data points. *N* = 6 per group. **P < 0.01 in *t*-tests. **h** Representative fluorescent images for TOMATO in the hypothalamus of male or female *POMC-CreER*^*T2*^*/Rosa26-tdTOMATO* littermates (tamoxifen injected at 11 weeks of age, and perfused at 16 weeks of age). **i** Quantifications of TOMATO-positive neurons (POMC neurons) in the hypothalamus of one out of five consecutive brain sections of male or female littermates (tamoxifen injected at 11 weeks of age, and perfused at 16 weeks of age). Data are presented as mean ± SEM with individual data points. *N* = 4 or 7 per group. **P < 0.01 in *t*-tests

We then compared the neural activities of TOMATO-labeled mature ARH POMC neurons (Fig. 1a) in male vs. female *POMC-CreER*^*T2*^*/Rosa26-tdTOMATO* mice. Interestingly, we observed that the firing rate in female POMC neurons was significantly higher, compared to male POMC neurons (Fig. 1b, c). In addition, the resting membrane potential in female POMC neurons was significantly depolarized, compared to that in male POMC neurons (Fig. 1b, d). In other words, female POMC neurons displayed significantly enhanced excitability, compared to male POMC neurons.

It has been reported that female mice express higher POMC mRNAs and protein products in the hypothalamus than male mice^30^. Consistently, here we detected significantly higher POMC mRNA levels in the hypothalamus of female mice than age-matched males (Fig. 1e). In addition, more β-endorphin (one POMC protein product)-positive neurons were observed in female ARH than in males (Fig. 1f,g). Increased number of POMC neurons (as revealed by β-endorphin immunostaining) may reflect higher POMC expression levels in each cell that exceed the detecting threshold by immunostaining; however, these results may also reflect the different POMC neuron numbers in female vs. male mice. Indeed, using *POMC-CreER*^*T2*^*/Rosa26-tdTOMATO* mice, in which all POMC neurons are labeled by TOMATO after tamoxifen induction (11 weeks), regardless of the expression levels of POMC gene, we found significantly more POMC neurons in the ARH of female mice than in male mice (Fig. 1h,i).

### TAp63 contributes to sex differences in body weight

**Fig. 2:**
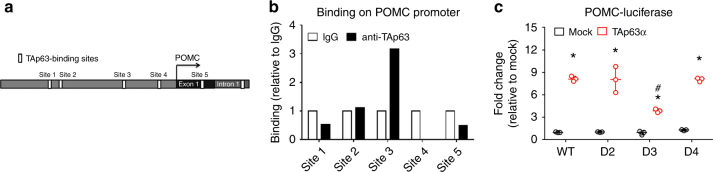
TAp63 stimulates POMC gene expression. **a** Five potential TAp63-binding sites on mouse POMC gene promoter. Site 1: −2011 to −1989; site 2: −1840 to −1818; site 3: −884 to −862; site 4: −155 to −135; site 5: 436–458, relative to the transcriptional start site. **b** CHIP assay using TAp63 antibody (or IgG as negative controls) to pull down the TAp63-binding sites from mouse hypothalamus. **c** Effects of TAp63α on POMC-luciferase activity in N46 cells. WT, pGL3b-POMC promoter; D2-4, pGL3b-POMC promoter with the deletion of sites 2, 3, or 4. Data are presented as box and wiskers showing minimal, maximal, and median values with individual data points. *N* = 3. *P < 0.05 vs. mock in the same POMC promoter; ^#^*P* < 0.05 vs. WT POMC promoter in response to the same TAp63 overexpression in two-way ANOVA analysis followed by post hoc Sidak tests

TAp63, a transcriptionally active variant of the transcription factor p63^31^, was recently identified as a novel defensive molecule against obesity, because mice with global deletion of TAp63 are more susceptible to diet-induced obesity^32^. We found five potential TAp63-binding sites on the promoter of the POMC gene, using Genomatix Region Miner release 3.2 (http://www.genomatix.de /index.html; Fig. 2a). We then performed chromatin-immunoprecipitation assay to show that TAp63 binds to site 3 on the POMC promoter in mouse hypothalamus (Fig. 2b). We then cloned the wild-type POMC promoter (WT), and made deletions in the sites 2, 3, or 4 (D2, D3 or D4 respectively), driving a luciferase reporter. We showed, in an immortalized mouse hypothalamic cell line (N46 cells), that overexpression of TAp63α (one C-terminal variant of TAp63) significantly stimulated the WT POMC-luciferase activity, and these stimulatory effects of TAp63α were not affected when sites 2 and 4 were deleted, but were substantially blunted when site 3 was deleted (Fig. 2c). Similar patterns were observed when the other two TAp63 C-terminal variants, namely TAp63β and TAp63γ, were overexpressed in cells (Supplementary Figure 2A-2B). Together, our data confirms that hypothalamic TAp63 directly binds to the POMC promoter, and overexpression of TAp63 is sufficient to activate POMC gene expression.

**Fig. 3:**
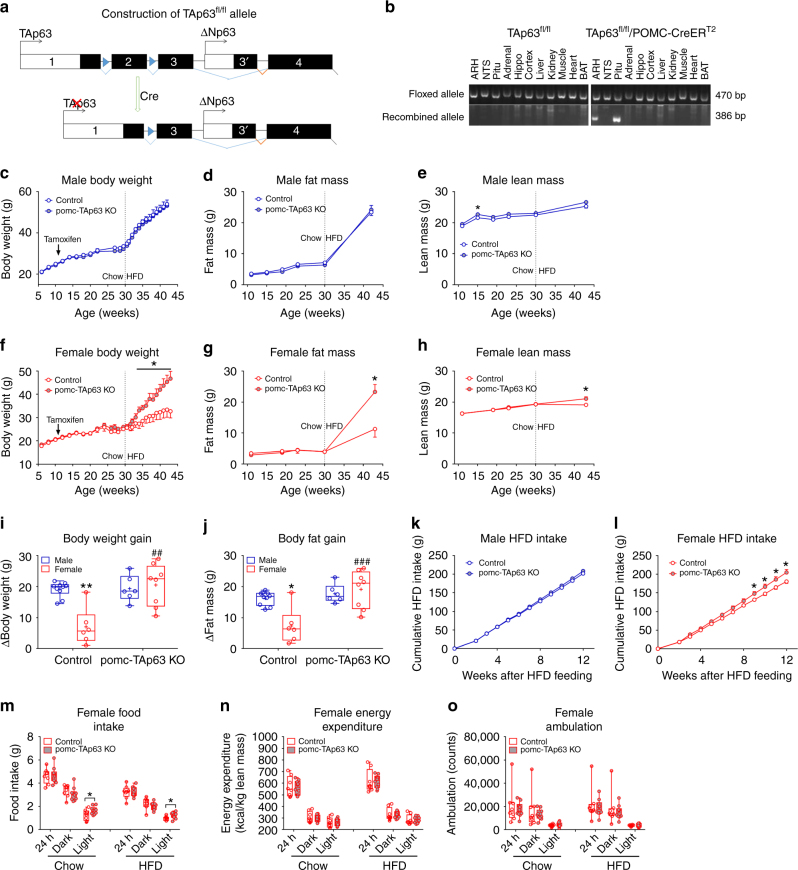
Deletion of TAp63 in POMC neurons diminishes sexual dimorphism in body weight responses to chronic HFD feeding. **a** Construction of TAp^fl/fl^ mouse allele. Two loxP sites flank exon 2 of the p63 gene and Cre-mediated recombination results in disruption of TAp63 expression; ΔNp63 expression starts from exon 3 and is therefore not affected by the recombination. **b** PCR detection of TAp^fl/fl^ allele and Cre-induced recombination in various tissues from the control (*TAp63*^*fl/fl*^) or the pomc-TAp63 KO (*TAp63*^*fl/fl*^*/POMC-CreER*^*T2*^) mice. Adrenal, adrenal gland; ARH, arcuate nucleus of the hypothalamus; BAT, brown adipose tissue; hippo, hippocampus; NTS, nucleus of the solitary tract; pitu, pituitary. **c**–**e** Body weight (**c**), fat mass (**d**), and lean mass (**e**) of male mice. Data are presented as mean ± SEM. *N* = 6 or 10 per group. *P < 0.05 in *t*-tests. **f**–**h** Body weight (**f**), fat mass (**g**), and lean mass (**h**) of female mice. Data are presented as mean ± SEM. *N* = 6 or 8 per group. *P < 0.05 in two-way ANOVA followed by post hoc Sidak tests. **i**–**j** Gains in body weight (**i**) and fat mass (**j**) during the 13-week HFD feeding. Data are presented as box and wiskers showing minimal, maximal, and median values with individual data points. *N* = 6–10 per group. *P < 0.05 or **P < 0.01 between male vs. female with the same genotype; ^##^*P* < 0.01 or ^###^*P* < 0.001 between the two genotypes within the same sex in two-way ANOVA followed by post hoc Sidak tests. **k**–**l** Cumulative HFD intake in male (**k**) and female (**l**) mice during the 13-week HFD feeding. Data are presented as mean ± SEM. *N* = 6 or 10 per group. *P < 0.05 in two-way ANOVA followed by post hoc Sidak tests. **m**–**o** Female control and pomc-TAp63 KO littermates (30 weeks of age) were adapted into the CLAMS metabolic cages and subjected to a 3-day chow 3-day HFD feeding protocol. Food intake (**m**), energy expenditure (normalized to lean mass, (**n**)), and ambulation (**o**) when fed chow or HFD. Data are presented as box and wiskers showing minimal, maximal, and median values with individual data points. *N* = 8 or 10 per group. *P < 0.05 between the two genotypes in *t*-tests

To examine the physiological functions of TAp63 in POMC neurons, we crossed *TAp63*^*fl/fl*^ mice^33^ with tamoxifen-inducible *POMC-CreER*^*T2*^ mice^34^ to generate pomc-TAp63 KO (*TAp63*^*fl/fl*^/*POMC-CreER*^*T2*^) mice. In the *TAp63*^*fl/fl*^ allele, two loxP sites flank exon 2 of the p63 gene, which is critical for TAp63 expression, while expression of another p63 gene variant (ΔNp63) starts from exon 3 and is therefore not affected (Fig. 3a, also see ref. 33). Tamoxifen induction (at 11 weeks of age) led to the recombination of the *TAp63*^*fl/fl*^ allele, specifically in adult POMC neurons in the ARH (Fig. 3b). Consistently, we used the RiboTag approach to confirm that TAp63 mRNAs in ARH POMC neurons were significantly reduced in both male and female pomc-TAp63 KO mice, compared to control mice, while the level of ΔNp63 was not altered (Supplementary Figure 3A-3B). Notably, we did not observe recombination of the *TAp63*^*fl/fl*^ allele in the nucleus of solitary tract (NTS) (Fig. 3b), where scarce POMC neurons are also located^35,36^, presumably due to the weak tamoxifen-induced Cre activity in this population. The *TAp63*^*fl/fl*^ allele was also recombined in the pituitary, which contains ACTH cells (Fig. 3b). However, there were no significant differences in pituitary levels of TAp63, ΔNp63, FSH β subunit, LH β subunit, or POMC (ACTH) between female control and pomc-TAp63 KO mice (Supplementary Figure 3C-3G); no difference in circulating ACTH was observed (Supplementary Figure 3H). Importantly, the levels of corticosterone were not altered in the pomc-TAp63 KO mice, compared to control mice, either at the basal condition or in response to the restraint stress (Supplementary Figure 3I). Thus, we suggest that the deletion of TAp63 in pituitary ACTH cells, if any, should not affect the hypothalamus-pituitary-gonad and the hypothalamus-pituitary-adrenal axes. Further, we examined the effects of tamoxifen injections on estrous cycles in female mice; while tamoxifen transiently disrupted the estrous cyclicity, it recovered in about 3 weeks after tamoxifen induction (Supplementary Figure 4). Thus, most of our studies were carried out at least 4 weeks after tamoxifen induction.

We monitored the body weight balance in male pomc-TAp63 KO mice and their littermate controls (*TAp63*^*fl/fl*^). Male pomc-TAp63 KO mice showed comparable body weight as their male controls, when fed on a regular chow diet (from weaning till 30 weeks of age; Fig. 3c). HFD feeding (from 30 weeks till 43 weeks of age) accelerated body weight gain in both pomc-TAp63 KO mice and control males, but no difference was observed between the two genotypes (Fig. 3c). No differences were observed in fat mass and lean mass, except that a modest increase in lean mass was observed in chow-fed pomc-TAp63 KO mice at 15 weeks of age (Fig. 3d, e). Thus, deletion of TAp63 in POMC neurons had none to minor effect on body weight balance and adiposity in male mice.

We also monitored the body weight gain in female pomc-TAp63 KO mice and their littermate controls. Similar to male mutants, pomc-TAp63 KO female mice showed comparable body weight, fat mass, and lean mass as their control females, when fed on the regular chow (Fig. 3f–h). Interestingly, when challenged with HFD feeding, pomc-TAp63 KO female mice gained significantly more body weight, compared to controls; at the end of 13-week of HFD feeding, pomc-TAp63 KO mice were 13 grams heavier than control mice (>30% of the control body weight; Fig. 3f). The body weight gain in female pomc-TAp63 KO mice was primarily due to a two-fold increase in fat mass, and to a less extent an increase in lean mass (Fig. 3g, h).

Given the sex-specific phenotypes observed in HFD-fed pomc-TAp63 KO mice, we analyzed the HFD-induced body weight gain and body fat gain in male vs. female mice with or without TAp63 deletion. Consistent with the notion that wild-type female rodents are more resistant to HFD-induced obesity, compared to their male counterparts^4–8,27^, we found that HFD-induced body weight gain and fat gain in female control mice were significantly lower than those in male control mice (Fig. 3i, j). Strikingly, pomc-TAp63 KO female mice gained as much body weight and fat as male mice (Fig. 3i, j). In other words, the sex differences in HFD-induced obesity were largely diminished by selective deletion of TAp63 in POMC neurons.

We then analyzed the food intake in chow or HFD-fed mice. We found that male mutants and their controls showed comparable food intake, regardless they were fed on chow or HFD (Fig. 3k S5A). Food intake in chow-fed pomc-TAp63 KO female mice were also similar to their chow-fed female controls (Supplementary Figure 5B). Importantly, HFD-fed pomc-TAp63 KO female mice showed increased food intake (Fig. 3l), although these changes only became significant toward the later phase of HFD feeding, which made it difficult to interpret their contributions to obesity. We then performed detailed analyses on energy balance in female pomc-TAp63 KO mice and their control littermates with comparable body weight, lean mass, and fat mass (Supplementary Figure 6A-6C). These mice were acclimated into the Comprehensive Lab Animal Monitoring System (CLAMS) and subjected to a 3-day chow 3-day HFD feeding protocol. While there was no significant changes in 24-h or dark cycle food intake (either chow or HFD) between the two genotypes, female pomc-TAp63 KO mice showed significantly increased light-cycle food intake (Fig. 2m), which has been associated with increased body weight gain^37,38^. On the other hand, we did not observe any significant changes in energy expenditure or ambulation between the two groups during these short chow or HFD-feeding periods (Fig. 2n, o).

### Estrogen-induced anorexigenic effects in female mice

**Fig. 4:**
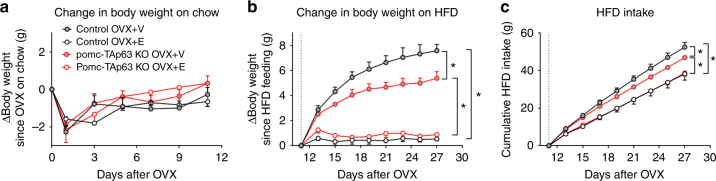
Selective deletion of TAp63 in POMC neurons fails to affect the estrogen-induced anorexigenic effects in female mice. **a**, **b** Changes in body weight of female control or pomc-TAp63 KO mice that received OVX+V or OVX+E treatment, followed by 11-day chow feeding (**a**) and 16-day HFD feeding (**b**). **c** Cumulative food intake during the HFD feeding period. Data are presented as mean ± SEM. *N* = 4 or 5 per group. *P < 0.05 in two-way repeated ANOVA followed by post hoc Sidak tests

The female-specific metabolic deficits observed in pomc-TAp63 KO mice suggested a potential interaction between the ovarian hormones and TAp63 functions in the regulation of energy balance. To test this, we subjected the female pomc-TAp63 KO mice and their control littermates to either ovariectomy (OVX+V) or OVX plus estrogen supplement (17β-estradiol, 0.5 µg per day lasting 90 days; OVX+E), followed by 11-day chow feeding and then 16-day HFD feeding. During the initial chow-feeding period, we did not observe significant differences in the body weight among all groups (Fig. 4a). During the following HFD feeding period, OVX+E treatment induced significant reductions in body weight and food intake, compared to OVX+V treatment, in both control and pomc-TAp63 KO mice (Fig. 4b, c). No significant differences were observed in body weight and HFD intake between OVX+E-treated controls and pomc-TAp63 KO mice (Fig. 4b, c). Thus, the lack of TAp63 in POMC neurons did not affect the anorexigenic effects of estrogens in female mice. Interestingly, we noted that OVX+V-treated pomc-TAp63 KO mice showed significantly less body weight gain and HFD intake, compared to OVX+V-treated control mice (Fig. 4b, c). Collectively, deletion of TAp63 from POMC neurons blunted the OVX-induced body weight gain in female mice, a phenotype associated with decreased food intake.

### Overexpression of TAp63 prevents DIO in male mice

**Fig. 5:**
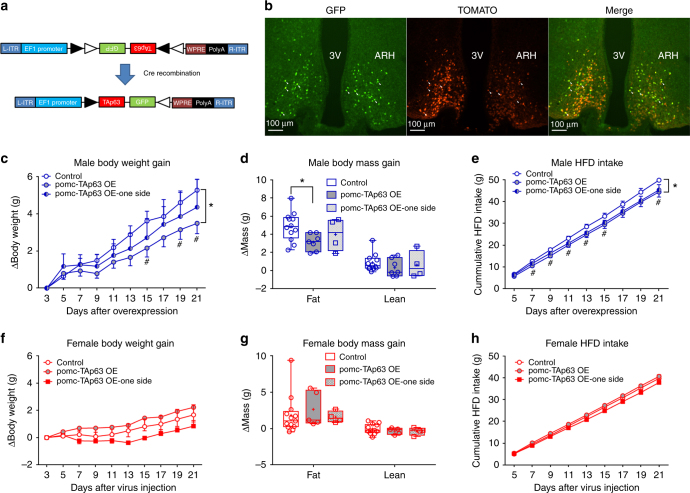
Selective overexpression of TAp63 in POMC neurons reduces the body weight of male HFD-fed mice. **a** Schematic construction of AAV vector, AAV-FLEX-TAp63-2A-GFP. **b** Immunofluorescent images showing GFP (left panel), TOMATO (middle panel), and merge (right panel) in POMC neurons of POMC-Cre/Rosa26-tdTOMATO mice receiving AAV-FLEX-TAp63-2A-GFP stereotaxically injected in the ARH bilaterally. The majority of GFP-labeled neurons are TOMATO-positive; only a few double-labeled neurons are pointed by arrowheads for a better view. 3 V, 3rd ventricle; ARH, arcuate nucleus of the hypothalamus. **c** Changes in body weight in HFD-fed male mice. Data are presented as mean ± SEM. *N* = 7 or 12 in control or OE group; *N* = 4 in one side group. *P < 0.05 between groups and ^#^*P* < 0.05 between the control and pomc-TAp63 OE groups at each time point in two-way ANOVA followed by post hoc Sidak tests. **d** Changes in the fat and lean mass in HFD-fed male mice. Data are presented as box and wiskers showing minimal, maximal, and median values with individual data points. *N* = 7 or 12 in control or OE group; *N* = 4 in one side group. *P < 0.05 in two-way ANOVA followed by post hoc Sidak tests. **e** Cumulative HFD intake in HFD-fed male mice. Data are presented as mean ± SEM. *N* = 7 or 12 in control or OE group; *N* = 4 in one side group. *P < 0.05 between groups and #*P* < 0.05 between the control and pomc-TAp63 OE groups at each time point in two-way ANOVA followed by post hoc Sidak tests. **f** Changes in body weight in HFD-fed female mice. Data are presented as mean ± SEM. *N* = 5 or 13 in control or OE group; *N* = 4 in one side group. **g** Changes in the fat and the lean mass in HFD-fed female mice. Data are presented as box and wiskers showing minimal, maximal, and median values with individual data points. *N* = 5 or 13 in control or OE group; *N* = 4 in one side group. **h** Cumulative HFD intake in HFD-fed female mice. Data are presented as mean ± SEM. *N* = 5 or 13 in control or OE group; *N* = 4 in one side group

To further investigate the function of TAp63 in POMC neurons, we tested the effects of TAp63 overexpression only in POMC neurons. To this end, we constructed an AAV vector, namely *AAV-EF1-FLEX-TAp63-2A-GFP*, which encodes *loxP*-flanked *TAp63* cDNA in an opposite orientation that overexpresses TAp63 and GFP only in Cre-positive cells (Fig. 5a). We stereotaxically injected this virus in the two sides (bilaterally) of ARH in *POMC-Cre* mice, resulting in TAp63 overexpression only in POMC neurons (referred as pomc-TAp63 OE; Fig. 5b).

When challenged with HFD feeding, male pomc-TAp63 OE mice showed significantly less body weight gain than controls (Fig. 5c), mainly reflected by reduced fat mass gain (Fig. 5d). Food intake of male pomc-TAp63 OE mice were significantly reduced, compared to controls (Fig. 5e). Interestingly, in male mice with only one side of the ARH hit by the virus (Supplementary Figure 7A), we observed partial and nonsignificant reductions in body weight, fat mass, and food intake (Fig. 5c–e). These data indicate that enhanced TAp63 expression in POMC neurons can partially prevent the development of DIO in male mice. In HFD-fed female mice, overexpression of TAp63 in POMC neurons did not appear to significantly change body weight, fat mass, and food intake (Fig. 5f–h). However, it is worth noting that we started HFD feeding 3 days after the virus injections. Thus, potential effects of overexpression in female mice may have been confounded by the short “incubation period” after the virus injections, together with the limited sample size in certain groups and big variations among individual animals. Further, TAp63 overexpression in POMC neurons did not significantly alter the body weight and the fat mass in chow-fed male or female mice, although a significant decrease in lean mass was observed in female mice with TAp63 overexpression (Supplementary Figure 7B-7E).

### Effects of TAp63 on POMC firing activities

**Fig. 6:**
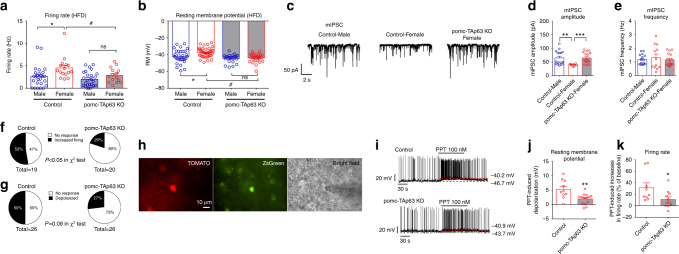
Selective deletion of TAp63 in POMC neurons impairs neural activities of POMC neurons. **a** Firing rate, recorded by the whole-cell patch clamp, in POMC neurons from male and female mice, with or without POMC-specific TAp63 deletion, that have been fed with HFD for 4 weeks. Data are presented as mean ± SEM with individual data points. *N* = 15–26 per group. *P < 0.05 between male and female with the same genotype; ^#^*P* < 0.05 between the two genotypes with the same sex in two-way ANOVA followed by post hoc Sidak tests. **b** Resting membrane potential, recorded by the perforated patch clamp, in POMC neurons from male and female mice, with or without POMC-specific TAp63 deletion, that have been fed with HFD for 4 weeks. Data are presented as mean ± SEM with individual data points. *N* = 22–39 per group. *P < 0.05 between male and female with the same genotype; ^#^*P* < 0.05 between the two genotypes with the same sex in two-way ANOVA followed by post hoc Sidak tests. **c** Typical mIPSC recorded from the POMC neurons of control male, control female, or pomc-TAp63 KO female mice that have been fed with HFD for 4 weeks. **d**, **e** Amplitude (**d**) and frequency (**e**) of mIPSC in POMC neurons. Data are presented as mean ± SEM with individual data points. *N* = 10–17 per group. **P < 0.01 or ***P < 0.001 in one-way ANOVA followed by Sidak tests. **f**, **g** Percentage of POMC neurons from control and pomc-TAp63 KO mice that were activated by PPT. PPT-induced activation was defined as either > 20% increases in firing rate (**f**) or by > 2 mV depolarization (**g**). **h** Fluorescence for TOMATO (left) and ZsGreen (middle), and bright-field illumination (right) of a recorded ERα-positive POMC neuron in a brain slice prepared from *ERα-ZsGreen/POMC-CreER*^*T2*^*/Rosa26-tdTOMATO* mouse with tamoxifen induction at 11 weeks of age. **i** Representative current clamp traces for PPT-induced responses in ERα-positive POMC neurons from control or pomc-TAp63 KO mice. **j**, **k** PPT-induced depolarization (**j**) and increases in firing rate (**k**) in ERα-positive POMC neurons from control or pomc-TAp63 KO mice. Data are presented as mean ± SEM. *N* = 10–14 per group. *P < 0.05 or **P < 0.01 in *t*-tests

In order to reveal the mechanisms by which TAp63 regulates POMC neurons, we analyzed the effects of TAp63 deletion on POMC neural activities in HFD-fed male and female mice. While the firing rate and resting membrane potential of POMC neurons were not significantly different between the male control and the male mutant mice, TAp63 deletion led to a significant reduction in firing rate and resting membrane potential in female POMC neurons (Fig. 6a, b). Importantly, the sex differences in firing rate and resting membrane potential of male vs. female POMC neuron were diminished by TAp63 deletion (Fig. 6a, b). Thus, these data indicate that TAp63 deletion diminished the sex differences in the excitability of POMC neurons.

We also compared the post-synaptic currents in male vs. female POMC neurons. No significant difference in glutamate-mediated miniature excitatory post-synaptic current (mEPSC) was observed (Supplementary Figure 8). Interestingly, we found that the amplitude of GABA-mediated miniature inhibitory post-synaptic current (mIPSC) was significantly lower in female POMC neurons than in male POMC neurons, while the mIPSC frequency was comparable between the sexes (Fig. 6c–e). More importantly, female POMC neurons lacking TAp63, displayed increased mIPSC amplitude to the level of male POMC neurons (Fig. 6c, d). Together, these data suggest that TAp63 in female POMC neurons suppresses their responsiveness to the inhibitory GABAergic inputs, a potential mechanism that contributes to enhanced excitability.

We have previously shown that ERα signals can trigger rapid activations in a portion of POMC neurons, which in turns produces anorexigenic effects in female mice^39,40^. Here we examined the effects of a selective ERα agonist, propyl pyrazole triol (PPT)^41^, on neural activities of POMC neurons from either control or pomc-TAp63 KO mice. While 10 out of 19 (53%) POMC neurons from control female mice were activated by PPT (>20% increase in firing rate), only 4 out of 20 (20%) POMC neurons from pomc-TAp63 KO female mice were activated by PPT (*P* < 0.05 in *χ*^2^-test, Fig. 6f). A similar trend was also observed when PPT-induced depolarization (>2 mV) was compared in control vs. pomc-TAp63 KO mice (*P* = 0.09 in *χ*^2^-test, Fig. 6g). Thus, these results indicate that TAp63 deletion reduced the percentage of POMC neurons that respond to the ERα agonist.

To further test the responsiveness of POMC neurons to estrogenic signals, we crossed a recently generated *ERα-ZsGreen*mouse allele^42^ with the control (*POMC-CreER*^*T2*^*/Rosa26-tdTOMATO*) mice and mutant (*POMC-CreER*^*T2*^*/TAp63*^*fl/fl*^*/Rosa26-tdTOMATO*) mice, and performed electrophysiological recordings in ERα-positive POMC neurons (double labeled by ZsGreen and TOMATO, Fig. 6h). We found that PTT activated all these ERα-positive POMC neurons, but the deletion of TAp63 significantly attenuated the PPT-induced depolarization and increases in firing rate (Fig. 6j, k). These results indicate that TAp63 deletion impaired the responsiveness of ERα-positive POMC neurons to the ERα agonist.

## Discussion

One major finding of the current study is that female POMC neurons have higher neural activities, compared to male POMC neurons, while such sexual dimorphism was not observed in most of the other neural populations we tested (e.g., AgRP/NPY neurons, SF1 neurons, and SIM1 neurons). Given the well-established anorexigenic nature of POMC neurons, these enhanced POMC neuron firing in female brains may contribute to the relatively lower body weight in female animals, compared to male counterparts. Interestingly, deletion of TAp63 in POMC neurons increased the susceptibility to DIO in female mice associated with decreased POMC neural activities; but the same deletion did not affect male mice. Although we could not fully exclude the possibility that TAp63 was not fully deleted from male POMC neurons, our results suggest that TAp63 in POMC neurons is one critical molecule that underlies, at least partly, sexual dimorphism in energy balance.

The mechanisms by which TAp63 in POMC neurons regulates energy balance, clearly involves its effects on POMC firing activities. We showed that TAp63 deletion resulted in decreased firing rate only in female POMC neurons, but not in male POMC neurons. The decreased POMC firing activities likely contribute to the increased food intake and increased susceptibility to DIO in these mutant mice. More importantly, while female POMC neurons had increased firing rates associated with decreased mISPC amplitude, compared to their male counterparts, this sexual dimorphism in POMC firing activities and mIPSC amplitude was abolished by TAp63 deletion. Thus, we suggest that TAp63 in female POMC neurons is required to suppress the responsiveness to GABAergic inputs and to maintain high firing rate of these neurons, which therefore distinguishes them from male POMC neurons. Although firing activities recorded from ex vivo brain slices may not fully reconstruct the impacts of physiological environments of the male vs. female animals on POMC neurons, our results supported the notion that TAp63 has cell-autonomous effects on POMC neuron activities in a sexually dimorphic fashion.

It is quite surprising that we detected increased body weight only during HFD feeding, but not during chow feeding. The lack of body weight gain in chow-fed pomc-TAp63 KO females suggest that redundant mechanisms may exist at the physiological condition to compensate for the impaired POMC neuron functions in these mutant mice. Similarly, loss of SIRT1 in POMC neurons, while resulting in robust body weight gain in HFD-fed female mice, causes no body weight gain in chow-fed females^43^. In addition, deletion of LKB1 in POMC neurons results in about 50% reduction in αMSH levels in female mice, but fails to affect the body weight balance^44^. Further, simultaneous deletion of the insulin receptor and the leptin receptor in POMC neurons leads to profound decreases in POMC gene expression, but does not affect the body weight in chow-fed mice^45^. These observations raise the possibility that impaired POMC neuron functions in chow-fed mice could be compensated by redundant mechanisms, and therefore fail to produce detectable body weight phenotypes. On the other hand, impaired POMC neuron functions combined with HFD feeding may be sufficient to cause energy imbalance, and thus result in detectable body weight gain. It is also worth noting that TAp63 has been shown to prevent skin aging by regulating cellular senescence and genomic stability^33^. Thus, the profound body weight gain during HFD feeding (starting from 30 weeks of age) may also be attributed, at least partly, to a combined impact of TAp63 deletion and aging.

The female sex hormone, estrogen, has been long believed to largely contribute to the sexual dimorphism in energy balance^9^. Indeed, we previously showed that ERα expressed by POMC neurons suppresses the food intake only in female mice, but not in male mice^39^. Thus, an important question is whether and how TAp63 interacts with estrogenic actions in POMC neurons. Our results suggest that the functional interactions between TAp63 and estrogen is complex. On one hand, certain functions of TAp63 and estrogen are independent of each other. For example, TAp63 deletion enhanced GABA-induced mIPSC in female POMC neurons. In addition, TAp63 can directly bind to the POMC promoter and stimulate its transcription. Notably, POMC expression in female hypothalamus is not affected by the estrous cycles^46^ or by ERα deletion^39^. Thus, both these functions of TAp63 do not appear to involve estrogenic actions. In parallel, TAp63 deletion in POMC neurons does not recapitulate some phenotypes seen in mice lacking ERα in POMC neurons, including changes in energy expenditure^39^. Thus, at least a portion of ERα actions in POMC neurons are unrelated to TAp63 functions.

On the other hand, TAp63 may be required for some estrogenic actions in POMC neurons. For example, the ERα-evoked neural activities in a portion of POMC neurons were reduced by TAp63 deletion. Notably, TAp63 deletion failed to significantly affect the effects of exogenous estrogen to reduce body weight and food intake in OVX females. Similar phenotypes were observed in female mice lacking ERα in POMC neurons, which develop hyperphagia^39^, but respond to exogenous estrogen with comparable reductions in body weight as their controls^40^. We speculate that this may be due to the compensations in other estrogen-responsive neurons, including those in the ventromedial hypothalamic nucleus^39,47,48^ and the medial amygdala^27^, which also mediate estrogenic actions to suppress the body weight. Interestingly, we found that female pomc-TAp63 KO mice were less sensitive to OVX-induced body weight gain, suggesting that TAp63 in POMC neurons is, at least partly, required for effects of endogenous ovarian hormones (estrogens and/or progesterone). These possibilities warrant future investigations.

In summary, we show that female POMC neurons display higher neural activities, compared to male counterparts. We further identified the transcription factor, TAp63, as one key molecule that underlies this sexually dimorphic feature of POMC neurons. Our results indicate that TAp63 regulates POMC neuron functions via both ERα-dependent and ERα-independent mechanisms. Importantly, TAp63, in POMC neurons, is required to prevent diet-induced obesity only in female mice, but not in male mice. Thus, these results provide important mechanistic insights onto the cellular and molecular basis for the sex differences in body weight control, and may facilitate the development of novel therapeutic interventions for obesity and associated metabolic disorders.

## Methods

### Mice

We crossed C57Bl6j mice (purchased from the mouse facility of Baylor College of Medicine) with NPY-GFP mice^49^ to generate NPY-GFP mice, which were used for electrophysiological recordings in AgRP/NPY neurons in the ARH. Further, SF1-Cre transgenic mice^26^ were bred with Rosa26-tdTOMATO mice to generate SF1-Cre/Rosa26-tdTOMATO mice. These mice were used for electrophysiological recordings in SF1 neurons in the VMH. In addition, SIM1-Cre transgenic mice^28^ were crossed with Rosa26-tdTOMATO mice to generate SIM1-Cre/Rosa26-tdTOMATO mice. These mice were used for electrophysiological recordings in SIM1 neurons in the PVH and the MeA.

In addition, tamoxifen-inducible *POMC-CreER*^*T2*^ transgenic allele^34^ was crossed with *TAp63*^*fl/fl*^ mice^33^ to generate *TAp63*^*fl/fl*^*/POMC-CreER*^*T2*^ (referred as pomc-TAp63 KO) and *TAp63*^*fl/fl*^ (referred as controls) littermates. In parallel, we also crossed the Rosa26-tdTOMATO and/or ERα-ZsGreen mouse alleles with *POMC-CreER*^*T2*^ mice or pomc-TAp63 KO to generate mice with POMC neurons labeled by TOMATO and/or ERα neurons labeled by ZsGreen; these mice were used for electrophysiological recordings. Similarly, we crossed the Rosa26-RiboTag mouse allele^50^ with *POMC-CreER*^*T2*^ mice or pomc-TAp63 KO to generate mice with POMC neurons expressing RiboTag; these mice were used for RiboTag studies described below. All these mice received tamoxifen treatment at 11 weeks of age (0.2 g^ kg-1^, i.p.), which induced Cre-recombinase activity in pomc-TAp63 KO mice. Electrophysiology and RiboTag experiments were performed at least 4 weeks after tamoxifen induction.

We made a Cre-dependent vector, *AAV8-EF1-FLEX-TAp63-2A-GFP*, to encode loxP-flanked *TAp63-2A-GFP* cDNA in the opposite orientation. This vector will overexpress TAp63 and GFP only in Cre-positive cells. *POMC-Cre* transgenic mice^51^ were crossed with C57BL/6N mice to produce *POMC-Cre* transgenic mice and WT littermates, and some *POMC-Cre* transgenic mice were crossed with *Rosa26-tdTOMATO* mice to generate *Rosa26-tdTOMATO/POMC-Cre* mice with POMC neurons labeled with TOMATO. All these mice (12 weeks of age) were anesthetized by isoflurane and received stereotaxic injections of *AAV-FLEX-TAp63-2A-GFP* virus into the ARH region (0.2 μl per side, 1.42 × 10^12^ GC ml^-1^), and TAp63 and GFP were only overexpressed in Cre-positive cells in the *POMC-Cre* mice (referred as pomc-TAp63 OE), but not in WT mice (referred as controls). The stereotaxic coordinates for the ARH were having anteroposteriority of −0.17 cm and laterality of 0.02 cm, relative to bregma, and the dorsoventrality of −0.59 cm, relative to the skull. After recovery from the surgery, these mice were fed either with chow or HFD, and the body weight and the food intake were measured every 2 days. At the end of the study, all the mice were perfused with 10% formalin. The brain sections were cut at 25 μm (1:5 series), and the sections were incubated in primary chicken anti-GFP antibody (1:5000; catalog GFP-1020, Aves Labs Inc.) overnight, followed by the goat–anti-chicken AlexaFluor 488 (1:250; catalog A11039, Invitrogen) for 1.5 h. Only mice with accurate targeting of the ARH (both sides) were included in the overexpression group for data analyses; some mice with only one side of the ARH targeted were included in a separate group for data analyses; mice without ARH targeted were excluded.

All mouse strains were backcrossed onto the C57BL/6N background for more than 12 generations.

Care of all animals and procedures was approved by Baylor College of Medicine Institutional Animal Care and Use Committees. Mice were housed in a temperature-controlled environment in groups of two to four at 22–24 °C, using a 12-h light and 12-h dark cycle. The mice were fed either standard chow diet (Cat#2916, Harlan Teklad) or HFD (60% fat, D12492, Research Diets), and water was provided ad libitum.

### Histology

Male and female *Rosa26-tdTOMATO/POMC-CreER*^*T2*^ mice received tamoxifen treatment at 11 weeks of age (0.2 g kg^−^^1^, i.p.). At 16 weeks of age, these mice were anesthetized with inhaled isoflurane, and quickly perfused with 10% formalin. The brain sections were cut at 25 μm and collected into five consecutive series. One series of the sections were subjected to direct visualization of TOMATO signals under a fluorescence microscope. Another series were subjected to β-endorphin immunohistochemical staining. Briefly, the brain sections were blocked (3% Goat–anti-rabbit serum for 1 h), incubated with rabbit anti-β-endorphin antibody (1:10,000; #H-022-33, Phoenix Peptide) on shaker at room temperature for 24 h and followed by biotinylated anti-rabbit secondary antibody (1:1,000; Vector) for 2 h. Sections were then incubated in the avidin-biotin complex (1:500, ABC; Vector Elite Kit) and incubated in 0.04% 3,3′-diaminobenzidine and 0.01% hydrogen peroxide. After dehydration through graded ethanol, the slides were then immersed in xylene and cover slipped. Images were analyzed using a bright-field microscope.

The numbers of TOMATO-positive or β-endorphin-positive neurons in the ARH were counted by blinded investigators in all brain sections containing the ARH (1/5 of the whole brain), and the total number was used to reflect the data value for that mouse. Several mice were included in each group for statistical analyses.

### Validation of TAp63^fl/fl^ recombination in POMC cells

Control and pomc-TAp63 KO mice (after tamoxifen inductions) were anesthetized with inhaled isoflurane and killed. Various tissues, as detailed in the figures, were collected. Genomic DNAs were extracted using the REDExtract-N-Amp Tissue PCR Kit (#XNATS; Sigma-Aldrich, St Louis, MO), followed by PCR amplification of the floxed or recombined alleles. Primer sequences were listed in Supplemental Table. RiboTag approach (described below) was also used to confirm the reduction of TAp63 mRNAs in POMC neurons.

### Body weight, body composition and food intake

Male and female pomc-TAp63 KO and control littermates were weaned on chow. The body weight was monitored weekly till the end of the study. Body composition was determined using quantitative magnetic resonance (QMR) at the time, as indicated in the figures. Mice were singly housed in order to measure the food intake in home cages.

### OVX+V and OVX+E

To examine whether TAp63 mediates the effect of estrogen on body weight control and glucose homeostasis, 12-week-old female pomc-TAp63 KO mice and control mice were anesthetized with inhaled isoflurane. As previously described^39,52^, bilateral ovariectomized (OVX) was performed followed by s.c. implantations of pellets containing 17β-estradiol (0.5 µg per day lasting for 90 days, OVX+E) or empty pellets (OVX+V). These pellets were purchased from Innovative Research of America. Mice were fed on regular chow for the first 11 days after the surgery and then fed on HFD for the following 16 days. Daily body weight and food intake were monitored from the 3rd day after the surgery.

### Hormone measurements

For the measurements of resting corticosterone, mice were rapidly decapitated at 9:00 am and the trunk blood was collected; to measure stress levels of corticosterone, mice were restrained for 60 min with a plastic restraint cone, and blood was collected from the tail vein. Plasma was obtained by centrifugation and assayed using a corticosterone EIA kit (900-097, Assay Designs, Ann Arbor, MI). Aliquots of plasma were also sent to Vanderbilt University Medical Center Hormone and Analytical Services Core to measure ACTH levels.

### Quantitative PCR in pituitary

After deep anesthesia, mice were decapitated, and the pituitaries were quickly collected and stored at –80 °C. Total RNAs were extracted using Ribozol (AMERESCO). Quantitative PCR (qPCR) was performed, as described previously^39^. Primer sequences were listed in Supplementary Table 1.

### CHIP-PCR

Hypothalamic samples from fed C56Bl6 mice were used to assess TAp63 binding at the POMC promoter. Anti-TAp63 antibody (618901, Biolegend) or IgG was used for immunoprecipitation. p63 binding sites were scanned within 4000 bp upstream of the 5′-UTR and intron 1, using Genomatix Region Miner release 3.2 (http://www.genomatix.de/index.html). All primers used for ChIP-PCR are listed in Supplemental Table. SYBR green PCR master mix (Applied Biosystems) were used for quantitative real-time PCR.

### Luciferase assay

A fragment of the mouse POMC promoter (-2229 to+589, relative to the transcription start site) was generated by PCR and was inserted into the PGL-3 basic vector (Promega), upstream of the luciferase reporter gene that express firefly. p53/p63 binding sites 2, 3, and 4 were deleted using QuikChange II Site-Directed Mutagenesis Kit (Agilent) from the WT promoter luciferase reporter, referred as D2, D3, and D4 POMC promoter. All the POMC promoter luciferase reporters (WT, D2, D3, and D4) were co-transfected with the pRL-TK plasmids that express Renilla together with *pcDNA3.1-TAp63β* or *pcDNA 3.1* vector as control to the neuronal cell line N46 (purchased from Cellutions) using Lipofectamine 2000 (GIBCO BRL), according to the manufacturer’s instructions. Twenty-four hours after transfection, the cells were lysed and assayed using dual-luciferase reagents (Promega). The promoter activity was measured by firefly luciferase levels and normalized to the constitutively expressed Renilla.

### RiboTag IP

Experiments involving female mice were performed at unknown estrous stage. Hypothalamic punches containing the entire ARH region (6-8 mg) were homogenized in 350 ul buffer, as described previously^50^. After centrifugation, 1 ul anti-HA antibody (MMS-101P, Covance) was added to 300 ul of the cleared lysate and incubated for 4 h at 4 °C. Remaining lysate was saved as input sample. After incubation, 50 ul of protein A/G agrose beads (Santa Cruz) was added and incubated overnight at 4 °C with rotation. Immunoprecipitates (IPs) were washed in high salt buffer and the RNA from inputs and IPs were extracted using Qiagen RNeasy Micro Plus kit (Qiagen), as described in the original protocol^50^. We sent aliquots of the RNA samples to Baylor Genomic and RNA Profiling (GARP) Core for quality control. Briefly, RNA samples were subjected to the Agilent Bioanalyzer 2100 to generate electropherograms. The two peaks of the 18S and 28S ribosomal RNAs were used to calculate the RNA integrity number (RIN) as a proxy for RNA quality. RIN higher than 8 indicates good quality. For IP sample, we used RT–qPCR to quantify transcripts of interest (e.g., TAp63 and ΔNp63, etc.) and the house-keeping gene (β-actin). We normalized the level of each transcript to the house-keeper level in the same IP sample to obtain a ratio, which was used to reflect the relative level of the transcript in that sample.

### Electrophysiology

Briefly, mice were deeply anesthetized with isoflurane and then decapitated at 9:00 am, and the entire brain was removed and immediately submerged in ice-cold sucrose-based cutting solution. Using a Microm HM 650 V vibratome (Thermo Scientific), the brains were cut into coronal slices (without trimming at other dimension) at the thickness of 250 µm each. Usually, 3–4 consecutive brain slices per mouse, ranging from Bregma −2.54 to −1.46 mm, contained the ARH and adjacent nuclei (the ventromedial hypothalamic nucleus, the dorsal medial hypothalamus, and the lateral hypothalamus) were used for recordings. The slices were recovered for 1 h at 34 °C and then maintained at room temperature in artificial cerebrospinal fluid (aCSF, adjusted to pH 7.3) containing (in mM) 126 NaCl, 2.5 KCl, 2.4 CaCl_2_, 1.2 NaH_2_PO_4_, 1.2 MgCl_2_, 11.1 glucose, and 21.4 NaHCO_3_ saturated with 95% O_2_ and 5% CO_2_ before recording^53^.

Slices were transferred to the recording chamber and allowed to equilibrate for at least 10 min before recording. The slices were superfused at 34 °C in oxygenated aCSF at a flow rate of 1.8-2 ml min^−1^. POMC neurons (or ERα-expressing POMC neurons) in the ARH were visualized using epifluorescence and IR-DIC imaging on an upright microscope (Eclipse FN-1, Nikon) equipped with a moveable stage (MP-285, Sutter Instrument). Patch pipettes with resistances of 3-5 MΩ were filled with intracellular solution (adjusted to pH 7.3) containing (in mM) 128 K gluconate, 10 KCl, 10 HEPES, 0.1 EGTA, 2 MgCl2, 0.3 Na-GTP, and 3 Mg-ATP^53^. Recordings were made using a MultiClamp 700B amplifier (Axon Instrument), sampled using Digidata 1440 A, and analyzed offline with pClamp 10.3 software (Axon Instrument). Series resistance was monitored during the recording, and the values were generally <10 MΩ and were not compensated. The liquid junction potential was+12.5 mV and was corrected after the experiment. Data were excluded if the series resistance increased dramatically during the experiment or without overshoot for action potential. Currents were amplified, filtered at 1 kHz, and digitized at 20 kHz. The whole-cell patch current clamp was engaged to test neural firing rate at the baseline and after puff of 100 nM PPT, as we did before^40,54^. The values for firing rate were averaged within 2 min bin at the baseline or after PPT treatment^53^. Some recordings were done using perforated patch. Briefly, the patch pipette solution for gramicidin perforated patch recording contained (in mM): KCl 150 and HEPES 10, pH 7.2 adjusted with Tris-OH. The gramicidin was first dissolved in DMSO in a stock solution of 60 mg mL^−1^ and then diluted to a final concentration of 30 µg mL^−^^1^. The gramicidin-free pipette solution was backfiled into the pipette first and the gramicidin-containing pipette solution was then added into the pipette.^55^ After 10–15 min of cell-attached formation, series resistance (Rs) decreased and stabilized at around 10 to 50 MΩ. The resting membrane was then recorded in the current clamp model. Action potential (AP) firing rate and resting membrane potential were measured using the clamp fit 10.3 software (Axon Instrument). For the miniature excitatory post-synaptic current (mEPSC) recordings, the internal recording solution contained: 125 mM CsCH3SO3; 10 mM CsCl; 5 mM NaCl, 2 mM MgCl_2_, 1 mM EGTA, 10 mM HEPES, 5 mM (Mg)ATP, and 0.3 mM (Na)GTP (pH 7.3 with NaOH). mEPSC in AgRP neurons was measured in the voltage clamp mode with a holding potential of −60 mV in the presence of 1 μM TTX and 50 μM bicuculline. The miniature inhibitory post-synaptic current (mIPSC) in POMC neurons was measured in the voltage clamp mode with a holding potential of −60 mV in the presence of 1 μM TTX and DAP-5 (30 μM; an NMDA receptor antagonist)^56^ and CNQX (30 μM; an NMDA receptor antagonist)^57^. Frequency and peak amplitude were measured using the Mini Analysis program (Synaptosoft, Inc.).

### Statistics

The minimal sample size was predetermined by the nature of experiments. For most of physiological readouts (body weight, food intake, etc.), at least 6 mice per group were included. For gene expression, 3–5 mice were included in each group. For electrophysiological studies, 20–40 neurons in each genotype or condition were included. The data are presented as mean ± SEM. Statistical analyses were performed using GraphPad Prism to evaluate normal distribution and variations within and among groups. Methods of statistical analyses were chosen based on the design of each experiment and are indicated in figure legends. *P* < 0.05 was considered to be statistically significant.

### Study approval

Care of all animals and procedures were conformed to the Guide for Care and Use of Laboratory Animals of the US National Institutes of Health and were approved by the Animal Subjects Committee of Baylor College of Medicine.

### Data availability

All relevant data are available from the authors.

## Electronic supplementary material

Supplementary Information
