# Supplementary material for: TAp63 contributes to sexual dimorphism in POMC neuron functions and energy homeostasis
Source: Nat Commun. 2018 Apr 18;9:1544. doi: 10.1038/s41467-018-03796-7 (PMC5906443; doi:10.1038/s41467-018-03796-7)
Supplement: Supplementary file 1 — Supplementary Information [file 41467_2018_3796_MOESM1_ESM.pdf]

TAp63 contributes to sexual dimorphism in POMC neuron functions and energy homeostasis

Chunmei Wang[1,6] , Yanlin He[1,6], Pingwen Xu[1,6], Yongjie Yang[1], Kenji Saito[1], Yan Xia[1], Xiaofeng Yan[1], Antentor Hinton Jr.[1], Chunling Yan[1], Hongfang Ding[1], Likai Yu[1], Gang Shu[1], Rajat Gupta[2], Qi Wu[1], Qingchun Tong[4], William R. Lagor[2], Elsa R. Flores[5] and Yong Xu[1,3]

Inventory

Supplementary Figure 1-8

Supplementary Figure 1 (related to Figure 1). Neural activities in other body weight-regulatory neural populations.

Supplementary Figure 2 (related to Figure 2). Effects of TAp63 on POMC expression.

Supplementary Figure 3 (related to Figure 3). Validation of pomc-TAp63 KO mice.

Supplementary Figure 4 (related to Figure 3). Estrous cycles in tamoxifen-injected female mice.

Supplementary Figure 5 (related to Figure 3). Food intake in chow-fed mice.

Supplementary Figure 6 (related to Figure 3). CLAMS study in female mice.

Supplementary Figure 7 (related to Figure 5). Effects of TAp63 overexpression.

Supplementary Figure 8 (related to Figure 6). mEPSC in male and female POMC neurons.

Supplemental Table 1. Primer sequences.

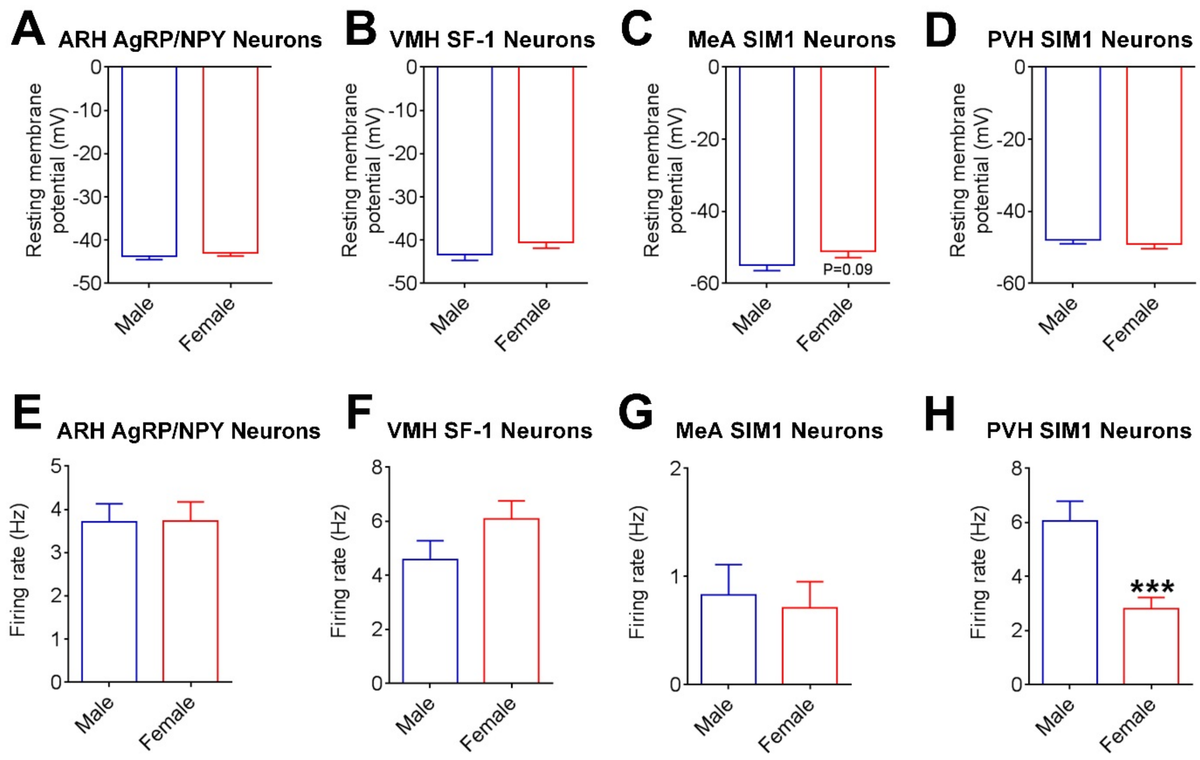

Supplementary Figure 1 (related to Figure 1). Neural activities in other body weight-regulatory neural populations. (A and E) Average resting membrane potential (A) and firing rate (E) in ARH AgRP/NPY neurons from male or female *NPY-EGFP* mice. Data are presented as mean±SEM. N=19 or 24 per group. (B and F) Average resting membrane potential (B) and firing rate (F) in VMH SF1 neurons from male or female *SF1-Cre/Rosa26-tdTOMATO* mice. Data are presented as mean±SEM. N=12 or 26 per group. (C and G) Average resting membrane potential (C) and firing rate (G) in MeA SIM1 neurons from male or female *SIM1-Cre/Rosa26-tdTOMATO* mice. Data are presented as mean±SEM. N=8 or 9 per group. (D and H) Average resting membrane potential (D) and firing rate (H) in PVH SIM1 neurons from male or female *SIM1-Cre/Rosa26-tdTOMATO* mice. Data are presented as mean±SEM. N=15 per group. *** P<0.001 in t-tests.

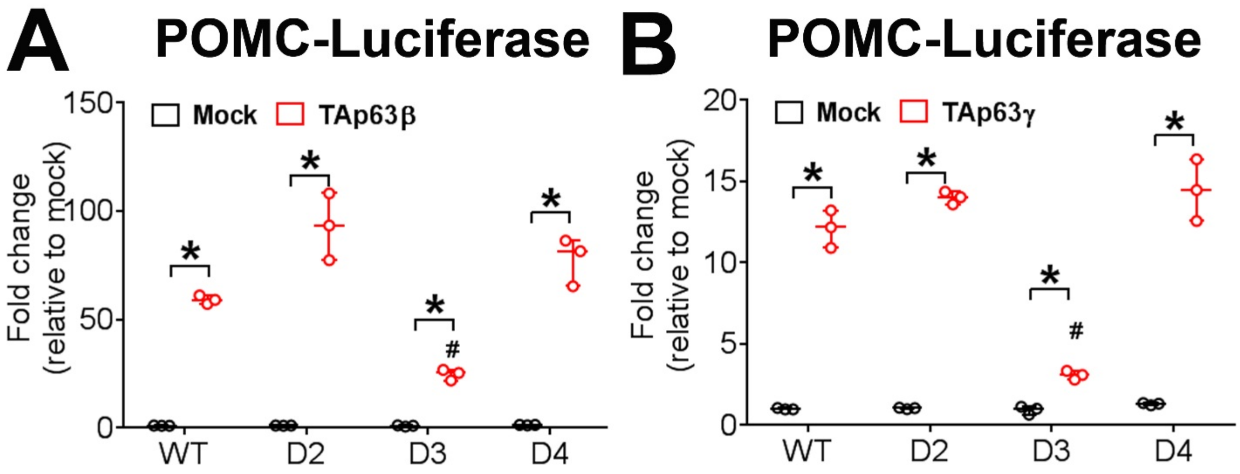

Supplementary Figure 2 (related to Figure 2). Effects of TAp63 on POMC expression. Effects of TAp63β (A) and TAp63γ (B) on POMC-luciferase activity in N46 cells. WT, pGL3b-POMC promoter; D2-4, pGL3b-POMC promoter with the deletion of site 2, 3 or 4. Data are presented as box and wiskers showing minimal, maximal and median values with individual data points. N=3. * $P<0.05$ vs. mock in the same POMC promoter; # $P<0.05$ vs WT POMC promoter in response to the same TAp63 overexpression in 2-way ANOVA analysis followed by post hoc Sidak tests.

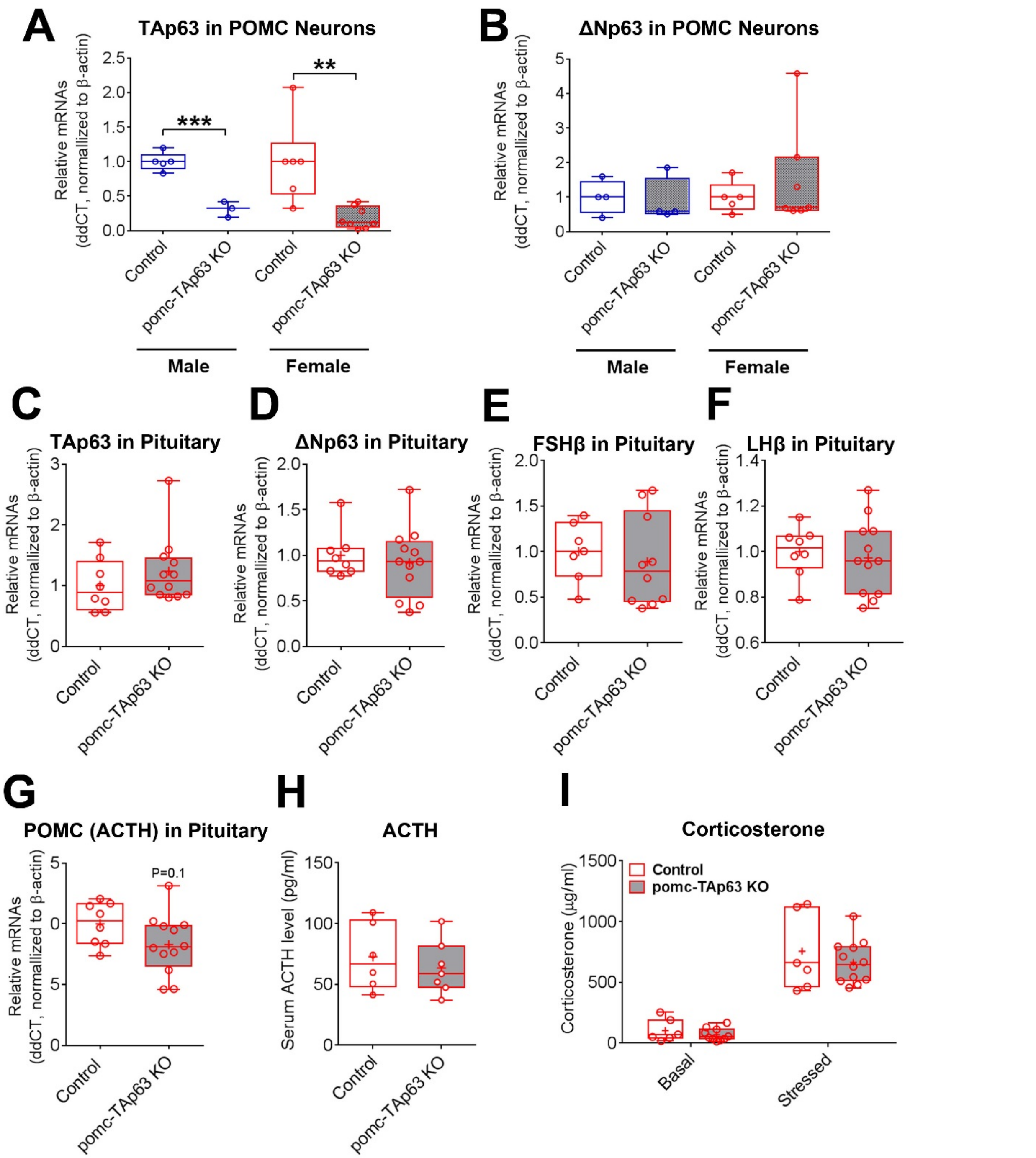

Supplementary Figure 3 (related to Figure 3). Validation of pomc-TAp63 KO mice. (A-B) RiboTag quantification of mRNAs of TAp63 (A) and ΔNp63 (B) in POMC neurons from HFD-fed male or female control (*RiboTag/TAp63$^{fl/+}$/POMC-CreER^{T2}*) or from pomc-TAp63 KO (*RiboTag/TAp63$^{fl/fl}$/POMC-CreER^{T2}*) mice at the age of 16 weeks of age. Data are presented as

mean±SEM with individual data points. N=3-8 per group. ** $P<0.01$ and *** $P<0.001$ in t-tests. (C-G) Regular real-time RT-PCR quantification of TAp63 (C), ΔNp63 (D), FSH β subunit (E), LH β subunit (F) and POMC/ACTH (G) mRNAs in the homogenized pituitaries of control or pomc-TAp63 KO female mice. Data are presented as box and wiskers showing minimal, maximal and median values with individual data points. N=8 or 12 per group. (H) Serum ACTH levels in control or pomc-TAp63 KO female mice. Data are presented as box and wiskers showing minimal, maximal and median values with individual data points. N=6 or 7 per group. (I) Serum corticosterone levels in control or pomc-TAp63 KO female mice at the basal condition or at stressed condition (30 min restraint). Data are presented as box and wiskers showing minimal, maximal and median values with individual data points. N=7-12 per group.

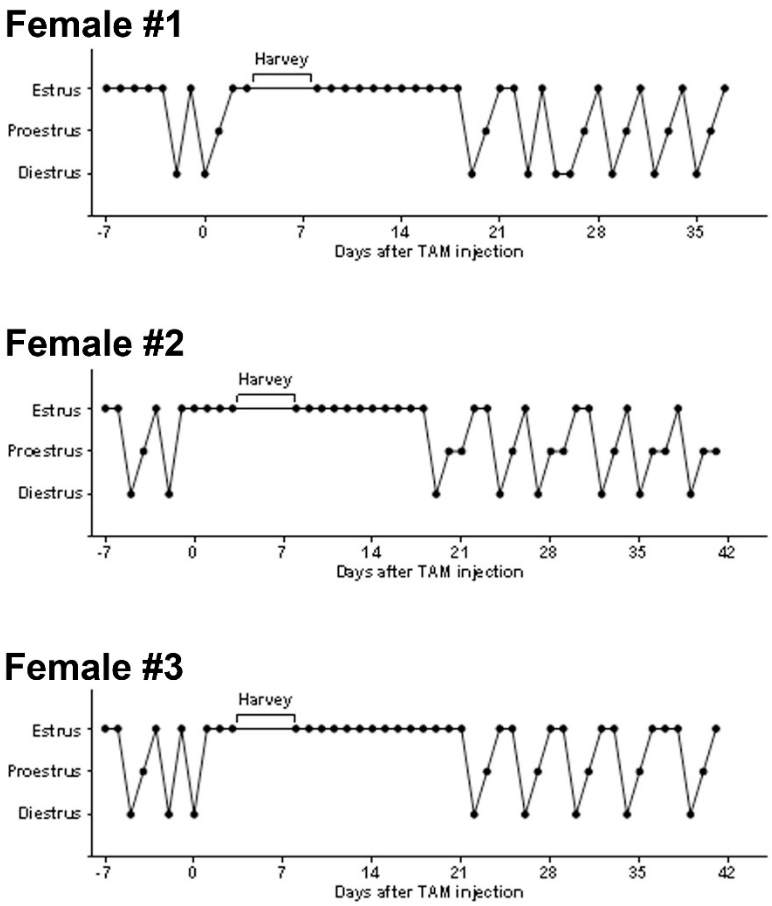

Supplementary Figure 4 (related to Figure 3). Estrous cycles in tamoxifen-injected female mice. Estrous stages of 3 female wild-type mice before and after they received tamoxifen injections (0.2 g kg^{-1}, i.p.) at 11 weeks of age. Note that 5-day data were missing due to the closure of the BCM animal facility during Hurricane Harvey.

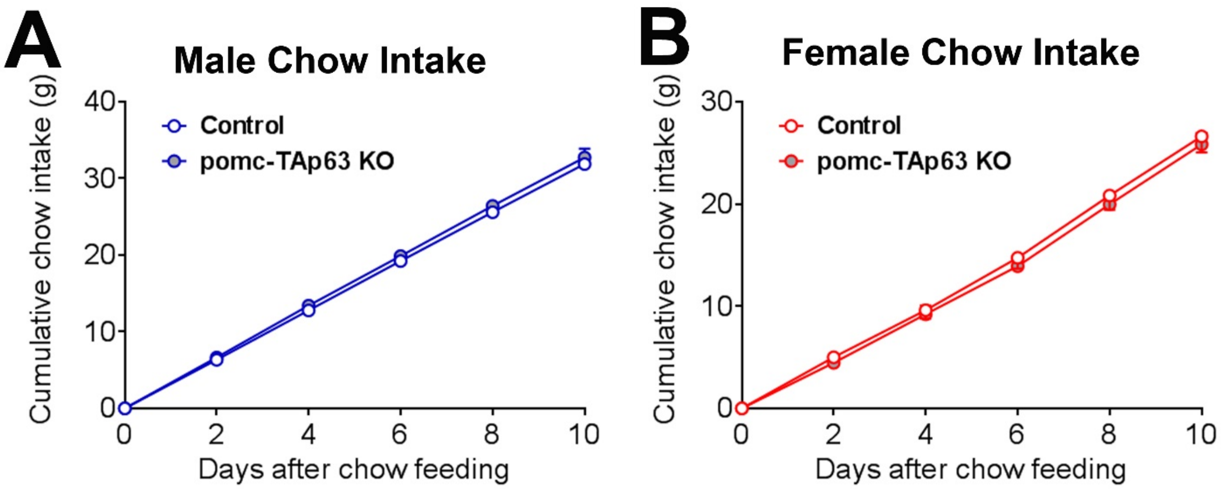

Supplementary Figure 5 (related to Figure 3). Food intake in chow-fed mice. (A) Cumulative chow intake in male mice. Data are presented as mean±SEM. N=5 or 9 per group. (B) Cumulative chow intake in female mice. Data are presented as mean±SEM. N=8 or 10 per group.

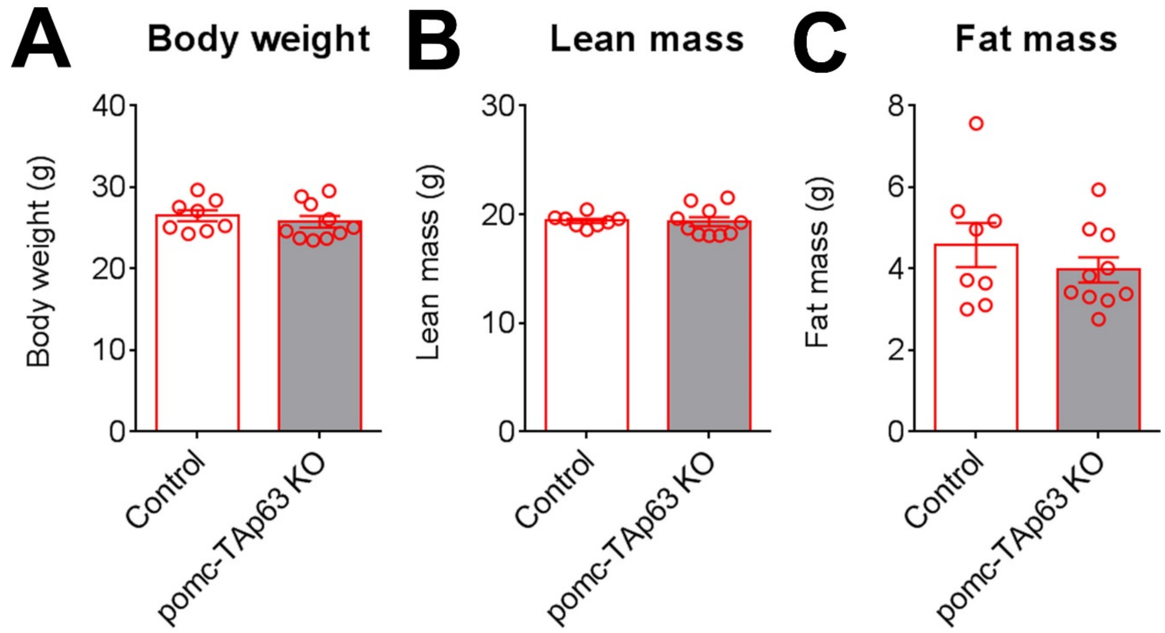

Supplementary Figure 6 (related to Figure 3). CLAMS study in female mice. Female control and pomc-TAp63 KO littermates (30 weeks of age) were adapted into the CLAMS metabolic cages and subjected to a 3-day-chow-3-day-HFD feeding protocol. (A-C) Body weight (A), lean mass (B) and fat mass (C) of mice when they entered the CLAMS. Data are presented as mean±SEM with individual data points. N=8 or 10 per group.

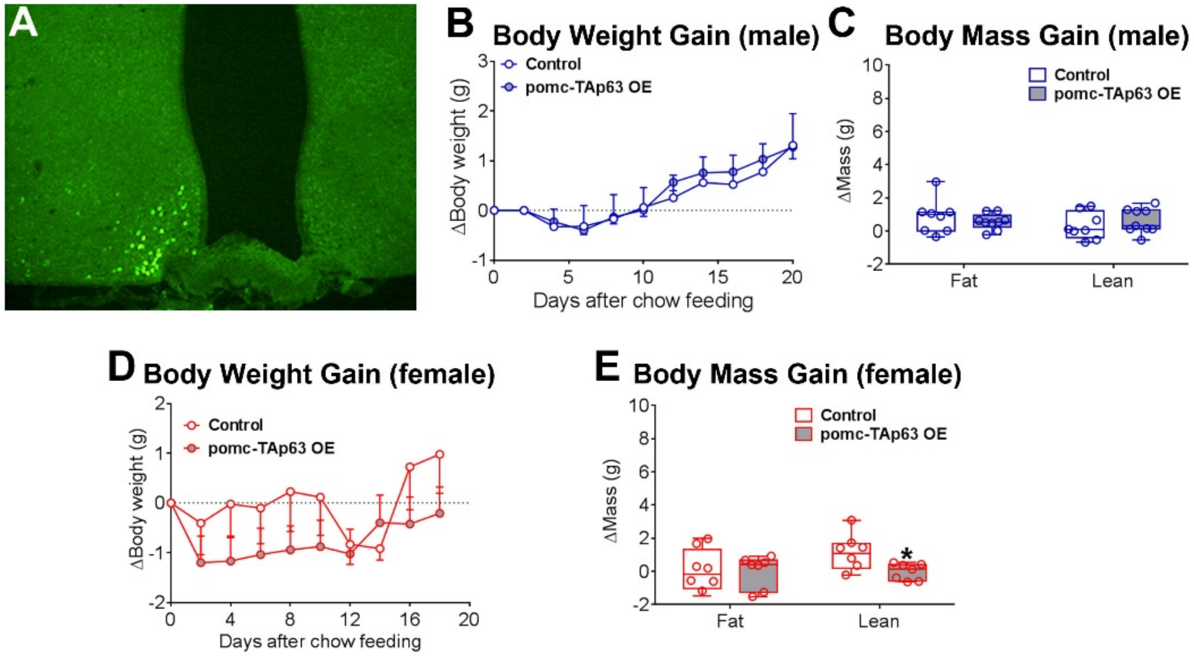

Supplementary Figure 7 (related to Figure 5). Effects of TAp63 overexpression. (A) A representative immunofluorescent image showing GFP only targeting one side of the ARH in POMC-Cre mice receiving AAV-FLEX-TAp63-2A-GFP stereotaxic injections. (B) Changes in body weight in chow-fed male control mice or mice with TAp63 overexpressed in POMC neurons. Data are presented as mean±SEM. N=8 or 9 in control or OE group. (C) Changes in fat and lean mass in male mice. Data are presented as box and wiskers showing minimal, maximal and median values with individual data points. N=8 or 9 per group. (D) Changes in body weight in chow-fed female control mice or mice with TAp63 overexpressed in POMC neurons. Data are presented as mean±SEM. N=7 in control or OE group. (E) Changes in fat and lean mass in male mice. Data are presented as box and wiskers showing minimal, maximal and median values with individual data points. N=7 per group.* $P<0.05$ between the two genotypes in t-tests.

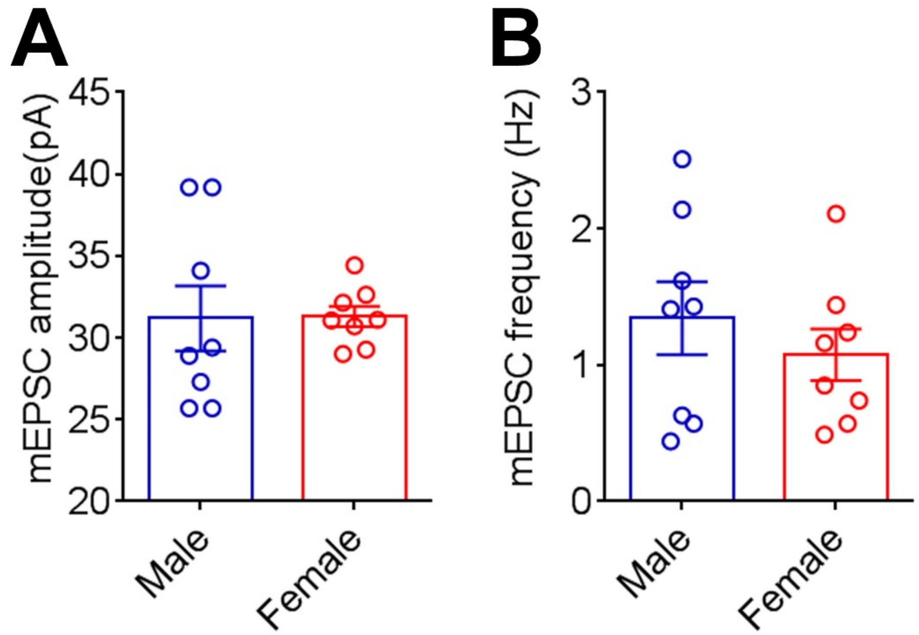

Supplementary Figure 8 (related to Figure 6). mEPSC in male and female POMC neurons. (A-B) Amplitude (A) and frequency (B) of mEPSC in POMC neurons from male vs. female *POMC-CreER*^{T2}*/Rosa26-tdTOMATO* mice with tamoxifen induction at 11 weeks of age followed by 4-week HFD feeding. Data are presented as mean±SEM with individual data point. N=8 per group.

Supplemental Table 1. Primer sequences.

Primer ID	Primer Sequence	Purpose
TAp63-fl F	CCACATAGCCATATCTGCC	Detecting the TAp63$^{fl/fl}$ allele
TAp63-fl R	TCGCCATAACTTCGTATAGC	
TAp63-KO F	CCTCACATCTGTCTCCTGACC	Detecting the recombined TAp63$^{fl/fl}$ alleles
TAp63-KO R	TTTTCGGAAGGTTCATCCAC	
POMC F	GAGGCCACTGAACATCTTTGTC	Detecting POMC mRNAs
POMC R	GCAGAGGCAAACAAGATTGG	
TAp63 F	CCCAGAGGTCTTCCAGCATA	Detecting TAp63 mRNAs
TAp63 R	TGCGGATACAATCCATGCTA	
Delta Np63 QF	AGGCTCTCAGAGGGGGTGGG	Detecting ΔNp63 mRNAs
Delta Np63 QR	ATTGAGTCTGGGCATTGTTTTCC	
FSHb-QF	TTCTGGTGCTGGAGAGCAAT	Detecting FSH β mRNAs
FSHb-QR	GCCGAGCTGGGTCCTTATAC	
LHb-QF	CTGAGCCCAAGTGTGGTGTG	Detecting LH β mRNAs
LHb-QR	GACCATGCTAGGACAGTAGCC	
b-actin F	ATGGAGGGGAATACAGCCC	Detecting β-actin mRNAs
b-actin R	TTCTTTGCAGCTCCTTCGTT	
POMC-luc-F	AAAACGCGTTTAAAAACCAAAAGGCCCCAGAAG	Constructing POMC promoter
POMC-luc-R	TTTCTCGAG TTATCACAATGCAACCACCCCAGA	
POMC D1F 2918	CCTGCTCCAGGCTATCAAAG	Detecting TAp63 binding on site 1
POMC D1R 3054	CCAGATCAGAATCAGGGTCAA	
POMC D2F 3030	GCCTTTGACCCTGATTCTGA	Detecting TAp63 binding on site 2
POMC D2R 3228	AGTGCACTGGCTGTTCTTCC	
POMC D3 F 4053	AGCGTCTAACTGGGGAGTGA	Detecting TAp63 binding on site 3
POMC D3 R 4198	CTGCAGTGGCATCTACCTGA	
POMC D4 F 4807	TCAGCGGGTCTGTGCTAAC	Detecting TAp63 binding on site 4
POMC D4 R 4998	TCCCTGTCGCTCTTCTCTCT	

POMC D5 F 5398	TTCTCCTTCCGATTGTTTGG	Detecting TAp63 binding on site 5
POMC D5 R 5565	TCTGCTAAGATGCGCAGAGA	
TAp63b virus F	ATTGCTAGCATGTCGCAGAGCACCCA	Constructing AAV-EF1-FLEX-TAp63-2A-GFP virus
TAp63b virus R	AAGACCGGT GACTTGCCAAATCCTGACAAT	
POMC-63-S1D-F	CTTTTTCCTGGTGACATCCTGGTAACAGGATATCCTGT	Constructing POMC-luc with site 1 deletion
POMC-63-S1D-R	ACAGGATATCCTGTTACCAGGATGTCACCAGGAAAAAG	
POMC-63-S2D-F	GAGCTGGAGTTTATAGACAGACGTGTTGAGAATTGAACCC	Constructing POMC-luc with site 2 deletion
POMC-63-S2D-R	GGGTTCAATTCTCAACACGTCTGTCTATAAACTCCAGCTC	
POMC-63-S3D-F	CCGACTGGTCTGGGCCAGCCACAGCT	Constructing POMC-luc with site 3 deletion
POMC-63-S3D-R	AGCTGTGGCTGGCCCAGACCAGTCGG	
POMC-63-S4D-F	CGCGCTTTCCAGGGCTCAGCCAGGAC	Constructing POMC-luc with site 4 deletion
POMC-63-S4D-R	GTCCTGGCTGAGCCCTGGAAAGCGCG	
POMC-63-S5D-F	GACTTGTATATCTTCTTTAAGGTTGGAAAGATAGCGGGAGAGAA	Constructing POMC-luc with site 5 deletion
POMC-63-S5D-R	TTCTCTCCCGCTATCTTTCCAACCTTAAAGAAGATATACAAGTC	
